# The solitary solutions for the stochastic fractional Chen Lee Liu model perturbed by multiplicative noise in optical fibers and plasma physics

**DOI:** 10.1038/s41598-024-60517-5

**Published:** 2024-05-07

**Authors:** Wael W. Mohammed, Naveed Iqbal, Rabeb Sidaoui, Monirah W. Alshammary

**Affiliations:** 1 Department of Mathematics, College of Science, University of Ha’il, 2440 Ha’il, Saudi Arabia; 2 Department of Mathematics, Faculty of Science, Mansoura University, Mansoura, 35516 Egypt

**Keywords:** Chen Lee Liu model, Mapping method, Conformable derivative, Stochastic exact solutions, Statistical physics, Applied mathematics

## Abstract

In this paper, we consider the stochastic fractional Chen Lee Liu model (SFCLLM). We apply the mapping method in order to get hyperbolic, elliptic, rational and trigonometric stochastic fractional solutions. These solutions are important for understanding some fundamentally complicated phenomena. The acquired solutions will be very helpful for applications such as fiber optics and plasma physics. Finally, we show how the conformable derivative order and stochastic term affect the exact solution of the SFCLLM.

## Introduction

Stochastic evolution equations are mathematical equations used to interpret the evolution of a system over time, taking into account both deterministic and random influences. They are widely used in various scientific disciplines, including physics, biology, and finance, to analyze complex systems that exhibit random behavior^1,2^. Stochastic evolution equations provide a powerful framework to study the dynamics of such systems, allowing scientists and researchers to better understand their behavior and make predictions. Because of the relevance of stochastic evolution equations, various methods have been developed to solve them, including He’s semi-inverse^3^, mapping method^4^, Jacobi elliptic function method^5^, Riccati equation mapping^6^, modified tanh–coth method^7,8^, modified fractional sub-equation method^9^, exp-function method^10^, and so on.

On the other hand, fractional evolution equations provide a powerful mathematical tool for modeling and understanding complex systems with long-range interactions. By incorporating fractional derivatives into the equations, these models can capture memory effects and interpolate between different classes of differential equations. The diverse applications of fractional evolution equations make them a valuable tool for researchers in various fields to analyze and simulate a wide range of phenomena, leading to a deeper understanding of complex systems^11–15^. Recently, there are numerous useful and effective techniques for solving these problems, such as modified simple equation method^16^, first integral method^17^, generalized Kudryashov method^18^, extended tanh–coth method^19–21^, exp-function method^22^, Jacobi elliptic function^23^, F-expansion technique^24^, and etc.

In this paper, we consider the stochastic fractional Chen Lee Liu model (SFCLLM) as follows^25^:

1 $$\begin{aligned} i\mathcal {G}_{t}+a\mathcal {D}_{xx}^{\alpha }\mathcal {G}+ib\left| \mathcal {G}\right| ^{2}\mathcal {D}_{x}^{\alpha }\mathcal {G}=i\rho \mathcal{G}\mathcal{B}_{t}, \end{aligned}$$

where $\mathcal {G}$ is the the normalized electric-field envelope, $\mathcal {D}_{x}^{\alpha }$ is a conformable fractional derivative (CFD), *a*, *b* and $\rho$ are positive constants, and $\mathcal {B}_{t}=\frac{\partial \mathcal {B}}{\partial t}$ is the derivative of the Brownain motion $\mathcal { B}(t).$

Due to the importance of the Chen Lee Liu model in fiber optics and plasma physics, many authors have used several methods in order to acquire the analytical solutions for this model such as Laplace Adomian decomposition method^26^, chirped W shaped optical solitons^27^, Darboux transformation^28^, modified extended tanh-expansion method^29^, Sardar sub-equation method^30^, Riccati–Bernoulli and generalized tanh methods^31^, ($G^{^{\prime }}/G,1/G)$-expansion approach^32^, extended direct algebraic method^33^, and modified Khater method^34^.

The purpose of this paper is to create the exact solutions of the SFCLLM (1). We apply the mapping method to produce a variety of solutions for instance hyperbolic, trigonometric, rational, and elliptic functions. Furthermore, we use Matlab program to create 2D and 3D graphs for some of the analytical solutions established in this paper to address the impact of the conformable fractional derivative and time-dependent coefficient on the acquired solutions of the SFCLLM (1).

The paper is organized as described below. In “Conformable derivative”, we define the CFD and describe some of its features. To attain the wave equation of the SFCLLM (1), we utilize an appropriate wave transformation in “Wave equation for SFCLLM”. In “The solutions of the SFCLLM”, we find the exact solutions of the SFCLLM (1) using the mapping method . In “Impacts of CD and noise”, we address the impact of the CFD and stochastic term on the attained solutions. Finally, the conclusion of the paper is introduced.

## Conformable derivative

Fractional calculus operators are an effective tool for modeling and evaluating complicated processes that cannot be effectively explained using regular integer-order calculus. Several types of fractional derivative operators have been suggested in the literature, including the Katugampola derivative, the Jumarie derivative, the Hadamard derivative, the Riemann–Liouville derivative, the Caputo derivative, and the Grünwald–Letnikov derivative^35–38^. In recent years, Khalil et al.^40^ proposed the conformable derivative (CD), which has features similar to Newton derivative. From here, let us define the CD for the function $\mathcal {P}:(0,\infty )\rightarrow \mathbb {R}$ of order $$\alpha \in (0,1]\ $$as follows:

$$\begin{aligned} \mathcal {D}_{x}^{\alpha }\mathcal {P}(x)=\lim _{\varepsilon \rightarrow 0} \frac{\mathcal {P}(x+\varepsilon x^{1-\alpha })-\mathcal {P}(x)}{\varepsilon }. \end{aligned}$$

The CD fulfills the next properties for any constant *a* and *b*:

- $$\mathcal {D}_{x}^{\alpha }[a\mathcal {P}_{1}(x)+b\mathcal {P}_{2}(x)]=a \mathcal {D}_{x}^{\alpha }\mathcal {P}_{1}(x)+b\mathcal {D}_{x}^{\alpha } \mathcal {P}_{2}(x),$$
- $$\mathcal {D}_{x}^{\alpha }[\mathcal {P}_{1}(x)\mathcal {P}_{2}(x)]= \mathcal {P}_{2}(x)\mathcal {D}_{x}^{\alpha }\mathcal {P}_{1}(x)+\mathcal {P} _{1}(x)\mathcal {D}_{x}^{\alpha }\mathcal {P}_{2}(x),$$
- $$\mathcal {D}_{x}^{\alpha }[a]=0,$$
- $$\mathcal {D}_{x}^{\alpha }[x^{b}]=bx^{b-\alpha }$$,
- $$\mathcal {D}_{x}^{\alpha }\mathcal {P}(x)=x^{1-\alpha }\frac{d\mathcal {P }}{dx}$$,
- $$\mathcal {D}_{x}^{\alpha }(\mathcal {P}_{1}\circ \mathcal {P} _{2})(x)=x^{1-\alpha }\mathcal {P}_{2}^{\prime }(x)\mathcal {P}_{1}^{\prime }( \mathcal {P}_{2}(x))$$.

## Wave equation for SFCLLM

To attain the wave equation of the SFCLLM (1), we utilize

2 $$\begin{aligned} \mathcal {G}(x,t)= & {} \varphi (\theta _{\alpha })e^{(i\psi _{\alpha }+\rho \mathcal {B}-\rho ^{2}t)}, \nonumber \\ \theta _{\alpha }= & {} \frac{\theta _{1}}{\alpha }x^{\alpha }+\theta _{2}t,\text { and }\psi _{\alpha }=\frac{\zeta _{1}}{\alpha }x^{\alpha }+\zeta _{2}t, \end{aligned}$$

where $\varphi$ is a real deterministic function. Plugging Eq. (2) into Eq. (1) and using

$$\begin{aligned} \frac{\partial \mathcal {G}}{\partial t}= & {} [\theta _{2}\varphi ^{\prime }+i\zeta _{2}\varphi \mathcal {+\rho }\varphi \mathcal {B}_{t}-\frac{1}{2}\rho ^{2}\varphi ]e^{(i\psi _{\alpha }+\rho \mathcal {B}-\rho ^{2}t)},\ \\ \ \mathcal {D}_{x}^{\alpha }\mathcal {G}= & {} (\theta _{1}\varphi ^{\prime }+i\zeta _{1}\varphi )e^{(i\psi _{\alpha }+\rho \mathcal {B}-\rho ^{2}t)},\ \\ \mathcal {D}_{xx}^{\alpha }\mathcal {G}= & {} [\theta _{1}^{2}\varphi ^{\prime \prime }+2i\zeta _{1}\theta _{1}\varphi ^{\prime }-\zeta _{1}^{2}\varphi ]e^{(i\psi _{\alpha }+\rho \mathcal {B}-\rho ^{2}t)},\ \end{aligned}$$

we get for imaginary part

3 $$\begin{aligned}{}[\theta _{2}+2a\zeta _{1}\theta _{1}]\varphi ^{\prime }+[\theta _{1}b]\varphi ^{2}\varphi ^{\prime }-\frac{1}{2}\rho ^{2}\varphi =0, \end{aligned}$$

and for real part

4 $$\begin{aligned} \varphi ^{\prime \prime }-A\varphi -B\varphi ^{3}e^{(2\rho \mathcal {B}-2\rho ^{2}t)}=0, \end{aligned}$$

where

5 $$\begin{aligned} A=\frac{(\zeta _{2}+a\zeta _{1}^{2})}{a\theta _{1}^{2}},\text { and }B=\frac{b \mathcal {\zeta }_{1}}{a\theta _{1}^{2}}\text { \ for }\theta _{1}\ne 0. \end{aligned}$$

Taking expectation $\mathbb {E[\cdot ]}$ on both sides of Eq. (4):

6 $$\begin{aligned} \varphi ^{\prime \prime }-A\varphi -B\varphi ^{3}e^{-2\rho ^{2}t}\mathbb {E[} e^{2\rho \mathcal {B}}]=0. \end{aligned}$$

Since $\mathcal {B}(t)$ is a Gaussain process, then

7 $$\begin{aligned} \varphi ^{\prime \prime }-A\varphi -B\varphi ^{3}=0. \end{aligned}$$

## The solutions of the SFCLLM

To find the solutions of Eq. (7), we use the mapping method, which is stated in^40^. Assuming the solutions of Eq. (7) take the form

8 $$\begin{aligned} \varphi (\theta _{\alpha })=\sum _{i=0}^{M}a_{i}(t)\mathcal {X}^{i}(\theta _{\alpha }), \end{aligned}$$

where $a_{i}(t)$ are undefined functions in *t* for $i=0,1,....,\ M$, and $\mathcal {X}$ is the solution of

9 $$\begin{aligned} \mathcal {X}^{\prime }=\sqrt{b_{1}\mathcal {X}^{4}+b_{2}\mathcal {X}^{2}+b_{3}}, \end{aligned}$$

where $b_{1},b_{2}$ and$\ b_{3}$ are real constants.

By balancing $\varphi ^{\prime \prime }$ with $\varphi ^{3}$ in Eq. (7), we can calculate *M* as

$$\begin{aligned} M+2=3M\Longrightarrow M=1. \end{aligned}$$

With $M=1$, Eq. (8) becomes

10 $$\begin{aligned} \varphi (\theta _{\alpha })=a_{0}+a_{1}\mathcal {X}(\theta _{\alpha }). \end{aligned}$$

Differentiating Eq. (10) two times and utilizing (9), we have

11 $$\begin{aligned} \varphi ^{\prime \prime }=a_{1}(b_{2}\mathcal {X}+2b_{1}\mathcal {X}^{3}). \end{aligned}$$

We obtain by substituting Eqs. (10) and (11) into Eq. (7)

$$\begin{aligned}{}[2a_{1}b_{1}-Ba_{1}^{3}]\mathcal {X}^{3}-3a_{0}a_{1}^{2}B\mathcal {X} ^{2}+[a_{1}b_{2}-Aa_{1}-3a_{0}^{2}a_{1}B]\mathcal {X}-[Ba_{0}^{3}+Aa_{0}]=0. \end{aligned}$$

For $$i=3,2,1,0,\ $$we put all coefficient of $\mathcal {X}^{i}$ equal zero to get

$$\begin{aligned}{} & {} 2a_{1}b_{1}-Ba_{1}^{3}=0, \\{} & {} \quad -3a_{0}a_{1}^{2}B=0, \\{} & {} \quad a_{1}b_{2}-Aa_{1}-3a_{0}^{2}a_{1}B=0, \end{aligned}$$

and

$$\begin{aligned} Ba_{0}^{3}+Aa_{0}=0. \end{aligned}$$

Solving these equations yields:

12 $$\begin{aligned} a_{0}=0,\ \ a_{1}=\pm \sqrt{\frac{2b_{1}}{B}},\ b_{2}=A. \end{aligned}$$

By using Eqs (2), (10) and (12), the solution of SFCLLM (1) is

13 $$\begin{aligned} \mathcal {G}(x,t)=\pm \sqrt{\frac{2b_{1}}{B}}\mathcal {X}(\theta _{\alpha })e^{(i\psi _{\alpha }+\rho \mathcal {B}-\rho ^{2}t)}. \end{aligned}$$

There are many sets depending on $b_{1},\ b_{2}$ and $b_{3}:$

Set 1: If $b_{1}=\tilde{n}^{2},\ b_{2}=-(1+\tilde{n}^{2})$ and$\ b_{3}=1,$ then $\mathcal {X}(\xi )=sn(\theta _{\alpha }).$ Therefore, by using Eq. (13), the solution of SFCLLM (1) is

14 $$\begin{aligned} \mathcal {G}(x,t)=\pm \tilde{n}\sqrt{\frac{2}{B}}sn(\theta _{\alpha })e^{(i\psi _{\alpha }+\rho \mathcal {B}-\rho ^{2}t)}\text {\ If }B>0. \end{aligned}$$

At $\tilde{n}\rightarrow 1,$ Eq. (14) becomes

15 $$\begin{aligned} \mathcal {G}(x,t)=\pm \sqrt{\frac{2}{B}}\tanh (\theta _{\alpha })e^{(i\psi _{\alpha }+\rho \mathcal {B}-\rho ^{2}t)}\text {\ \ If }B>0. \end{aligned}$$

Set 2: If $b_{1}=1,\ b_{2}=2\tilde{n}^{2}-1$ and$\ b_{3}=-\tilde{n} ^{2}(1-\tilde{n}^{2}),$ then $\mathcal {X}(\theta _{\alpha })=ds(\theta _{\alpha }).$ Consequently, the solution of SFCLLM (1), by using Eq. ( 13), is

16 $$\begin{aligned} \mathcal {G}(x,t)=\pm \sqrt{\frac{2}{B}}ds(\theta _{\alpha })e^{(i\psi _{\alpha }+\rho \mathcal {B}-\rho ^{2}t)}\text {\ If }B>0. \end{aligned}$$

When $\tilde{n}\rightarrow 1,$ Eq. (16) is typically

17 $$\begin{aligned} \mathcal {G}(x,t)=\pm \sqrt{\frac{2}{B}}\text {csch}(\theta _{\alpha })e^{(i\psi _{\alpha }+\rho \mathcal {B}-\rho ^{2}t)}\text {\ If }B>0. \end{aligned}$$

At $\tilde{n}\rightarrow 0,$ Eq. (16) tends to

18 $$\begin{aligned} \mathcal {G}(x,t)=\pm \sqrt{\frac{2}{B}}\csc (\theta _{\alpha })e^{(i\psi _{\alpha }+\rho \mathcal {B}-\rho ^{2}t)}\text {\ If }B>0. \end{aligned}$$

Set 3: If $b_{1}=-\tilde{n}^{2},\ b_{2}=2\tilde{n}^{2}-1$ and$\ b_{3}=1-\tilde{n}^{2},$ then $\mathcal {X}(\theta _{\alpha })=cn(\theta _{\alpha })$. Consequently, the solution of SFCLLM (1) is

19 $$\begin{aligned} \mathcal {G}(x,t)=\pm \tilde{n}\sqrt{\frac{-2}{B}}[cn(\theta _{\alpha })]e^{(i\psi _{\alpha }+\rho \mathcal {B}-\rho ^{2}t)}\text {\ If }B<0. \end{aligned}$$

When $\tilde{n}\rightarrow 1,$ Eq. (19) is typically

20 $$\begin{aligned} \mathcal {G}(x,t)=\pm \sqrt{\frac{-2}{B}}[\text {sech}(\theta _{\alpha })]e^{(i\psi _{\alpha }+\rho \mathcal {B}-\rho ^{2}t)}\text {\ If }B<0. \end{aligned}$$

Set 4: If $b_{1}=\frac{\tilde{n}^{2}}{4},\ b_{2}=\frac{(\tilde{n} ^{2}-2)}{2}$ and$\ b_{3}=\frac{1}{4},$ then $\mathcal {X}(\theta _{\alpha })= \frac{sn(\theta _{\alpha })}{1+dn(\theta _{\alpha })}.$ Consequently, the solution of SFCLLM (1) is

21 $$\begin{aligned} \mathcal {G}(x,t)=\pm \frac{\tilde{n}}{2}\sqrt{\frac{2}{B}}[\frac{sn(\theta _{\alpha })}{1+dn(\theta _{\alpha })}]e^{(i\psi _{\alpha }+\rho \mathcal {B} -\rho ^{2}t)}\text {\ If }B>0. \end{aligned}$$

At $\tilde{n}\rightarrow 1,$ Eq. (21) tends to

22 $$\begin{aligned} \mathcal {G}(x,t)=\pm \frac{1}{2}\sqrt{\frac{2}{B}}[\frac{\tanh (\theta _{\alpha })}{1+\text {sech}(\theta _{\alpha })}]e^{(i\psi _{\alpha }+\rho \mathcal {B}-\rho ^{2}t)}\text {\ If }B>0. \end{aligned}$$

Set 5: If $b_{1}=\frac{(1-\tilde{n}^{2})^{2}}{4},\ b_{2}=\frac{(1- \tilde{n}^{2})^{2}}{2}$ and$\ b_{3}=\frac{1}{4},$ then $\mathcal {X}(\theta _{\alpha })=\frac{sn(\theta _{\alpha })}{dn(\theta _{\alpha })+cn(\theta _{\alpha })}.$ Therefore, the solution of SFCLLM (1) is

23 $$\begin{aligned} \mathcal {G}(x,t)=\pm \frac{(1-\tilde{n}^{2})}{2}\sqrt{\frac{2}{B}}[\frac{ sn(\theta _{\alpha })}{dn(\theta _{\alpha })+cn(\theta _{\alpha })} ]e^{(i\psi _{\alpha }+\rho \mathcal {B}-\rho ^{2}t)}\text {\ If }B>0. \end{aligned}$$

If $\tilde{n}\rightarrow 0,$ then Eq. (23) is typically

24 $$\begin{aligned} \mathcal {G}(x,t)=\pm \frac{1}{2}\sqrt{\frac{2}{B}}[\frac{\sin (\theta _{\alpha })}{1+\cos (\theta _{\alpha })}]e^{(i\psi _{\alpha }+\rho \mathcal {B }-\rho ^{2}t)}\text {\ If }B>0. \end{aligned}$$

Set 6: If $b_{1}=\frac{1-\tilde{n}^{2}}{4},\ b_{2}=\frac{(1-\tilde{n }^{2})}{2}$ and$\ b_{3}=\frac{1-\tilde{n}^{2}}{4},$ then $\mathcal {X}(\theta _{\alpha })=\frac{cn(\theta _{\alpha })}{1+sn(\theta _{\alpha })}$. Consequently, the solution of SFCLLM (1) is

25 $$\begin{aligned} \mathcal {G}(x,t)=\pm \frac{1}{2}\sqrt{\frac{2(1-\tilde{n}^{2})}{B}}[\frac{ cn(\theta _{\alpha })}{1+sn(\theta _{\alpha })}]e^{(i\psi _{\alpha }+\rho \mathcal {B}-\rho ^{2}t)}\text {\ If }B>0. \end{aligned}$$

At $\tilde{n}\rightarrow 0,$ Eq. (25) turns to

26 $$\begin{aligned} \mathcal {G}(x,t)=\pm \frac{1}{2}\sqrt{\frac{2}{B}}[\frac{\cos (\theta _{\alpha })}{1+\sin (\theta _{\alpha })}]e^{(i\psi _{\alpha }+\rho \mathcal {B }-\rho ^{2}t)}\text {\ If }B>0. \end{aligned}$$

Set 7: If $b_{1}=1,\ b_{2}=0$ and$\ b_{3}=0,$ then $$\mathcal {X} (\theta _{\alpha })=\frac{c}{\theta _{\alpha }}.\ $$Therefore, the solution of SFCLLM (1) is

27 $$\begin{aligned} \mathcal {G}(x,t)=\pm \sqrt{\frac{2}{B}}[\frac{c}{\theta _{\alpha }} ]e^{(i\psi _{\alpha }+\rho \mathcal {B}-\rho ^{2}t)}\text {\ If }B>0. \end{aligned}$$

Set 8: If $b_{1}=1,\ b_{2}=2-\tilde{n}^{2}$ and$\ b_{3}=(1-\tilde{n} ^{2}),$ then $\mathcal {X}(\theta _{\alpha })=cs(\theta _{\alpha }).$ Therefore, the solution of SFCLLM (1) is

28 $$\begin{aligned} \mathcal {G}(x,t)=\pm \sqrt{\frac{2}{B}}cs(\theta _{\alpha })e^{(i\psi _{\alpha }+\rho \mathcal {B}-\rho ^{2}t)}\text {\ If }B>0. \end{aligned}$$

At $\tilde{n}\rightarrow 1,$ Eq. (28) is typically

29 $$\begin{aligned} \mathcal {G}(x,t)=\pm \sqrt{\frac{2}{B}}\text {csch}(\theta _{\alpha })e^{(i\psi _{\alpha }+\rho \mathcal {B}-\rho ^{2}t)}\text {\ If }B>0. \end{aligned}$$

If $\tilde{n}\rightarrow 0,$ then Eq. (28) becomes

30 $$\begin{aligned} \mathcal {G}(x,t)=\pm \sqrt{\frac{2}{B}}\cot (\theta _{\alpha })e^{(i\psi _{\alpha }+\rho \mathcal {B}-\rho ^{2}t)}\text {\ If }B>0. \end{aligned}$$

Set 9: If $b_{1}=\frac{-1}{4},\ b_{2}=\frac{\tilde{n}^{2}+1}{2}$ and $\ b_{3}=\frac{-(1-\tilde{n}^{2})^{2}}{2},$ then $$\mathcal {X}(\theta _{\alpha })=\tilde{n}cn(\theta _{\alpha })+dn(\theta _{\alpha }).\ $$ Therefore, the solution of SFCLLM (1) is

31 $$\begin{aligned} \mathcal {G}(x,t)=\pm \frac{1}{2}\sqrt{\frac{-2}{B}}[\tilde{n}cn(\theta _{\alpha })+dn(\theta _{\alpha })]e^{(i\psi _{\alpha }+\rho \mathcal {B}-\rho ^{2}t)}\text {\ If }B<0. \end{aligned}$$

When $\tilde{n}\rightarrow 1,$ Eq. (31) tends to Eq. (20 ).

Set 10: If $b_{1}=\frac{\tilde{n}^{2}-1}{4},\ b_{2}=\frac{\tilde{n} ^{2}+1}{2}$ and$\ b_{3}=\frac{\tilde{n}^{2}-1}{4},$ then $$\mathcal {X}(\theta _{\alpha })=\frac{dn(\theta _{\alpha })}{1+sn(\theta _{\alpha })}.\ $$Hence, the solution of SFCLLM (1) is

32 $$\begin{aligned} \mathcal {G}(x,t)=\pm \frac{1}{2}\sqrt{\frac{2(\tilde{n}^{2}-1)}{B}}[\frac{ dn(\theta _{\alpha })}{1+sn(\theta _{\alpha })}]e^{(i\psi _{\alpha }+\rho \mathcal {B}-\rho ^{2}t)}\text {\ If }B<0. \end{aligned}$$

When $\tilde{n}\rightarrow 0,$ Eq. (32) is typically

33 $$\begin{aligned} \mathcal {G}(x,t)=\pm \frac{1}{2}\sqrt{\frac{-2}{B}}[\frac{1}{1+\sin (\theta _{\alpha })}]e^{(i\psi _{\alpha }+\rho \mathcal {B}-\rho ^{2}t)}\text {\ If } B<0. \end{aligned}$$

Set 11: If $b_{1}=-1,\ b_{2}=2-\tilde{n}^{2}$ and$\ b_{3}=\tilde{n} ^{2}-1,$ then $$\mathcal {X}(\theta _{\alpha })=dn(\theta _{\alpha }).\ $$ Therefore, the solution of SFCLLM (1) is

34 $$\begin{aligned} \mathcal {G}(x,t)=\pm \sqrt{\frac{-2}{B}}[dn(\theta _{\alpha })]e^{(i\psi _{\alpha }+\rho \mathcal {B}-\rho ^{2}t)}\text {\ If }B<0. \end{aligned}$$

If $\tilde{n}\rightarrow 1,$ then Eq. (34) turns to Eq. (20).

## Impacts of CD and noise

### Impacts of CD

**Figure 1:**
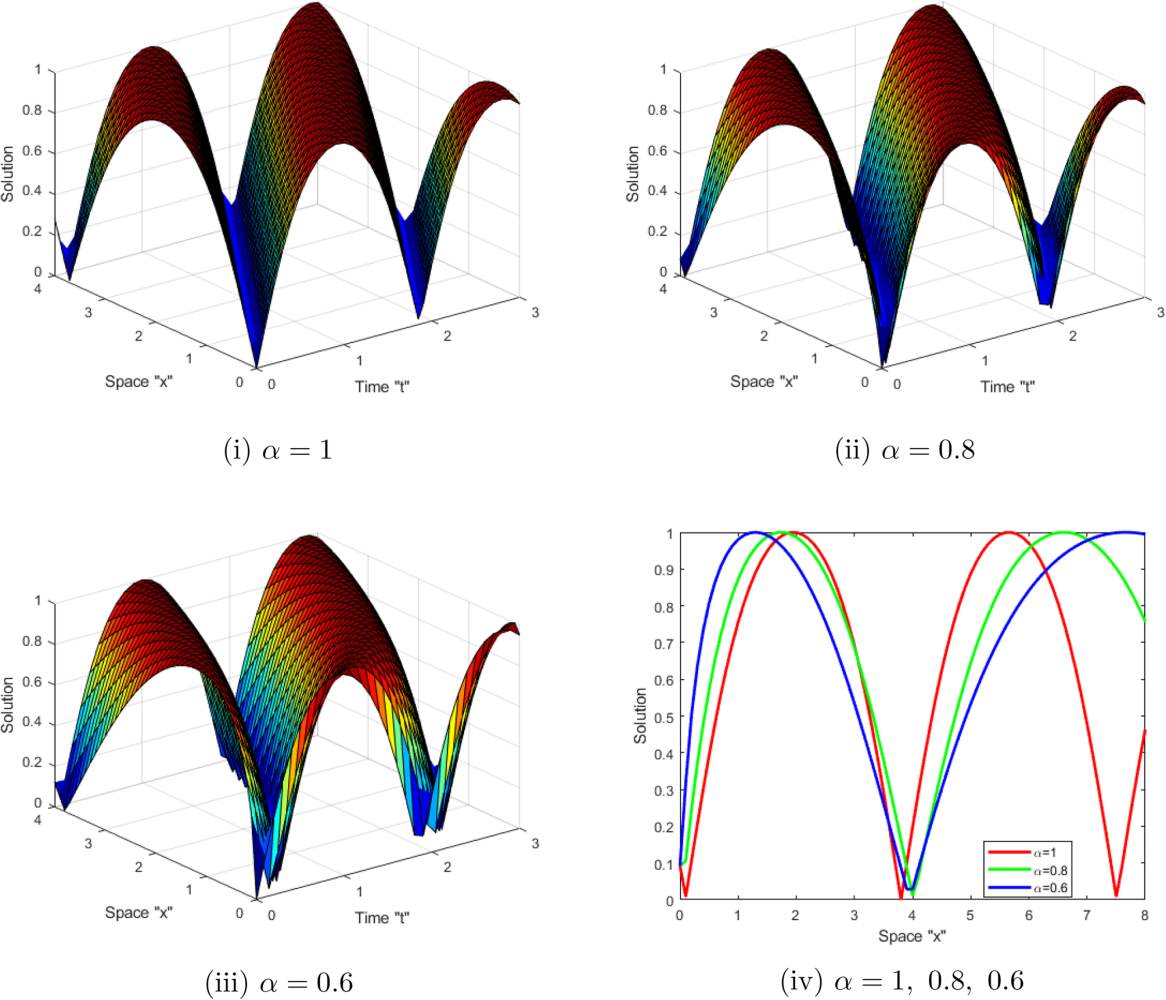
(**i–iii**) 3D-profile of the periodic solution $\left| \mathcal {G}(x,t)\right|$ described in Eq. (14) with $\alpha =1,\ 0.8,\ 0.6$ (**iv**) depict 2D-profile of Eq. (14) with various $\alpha$.

**Figure 2:**
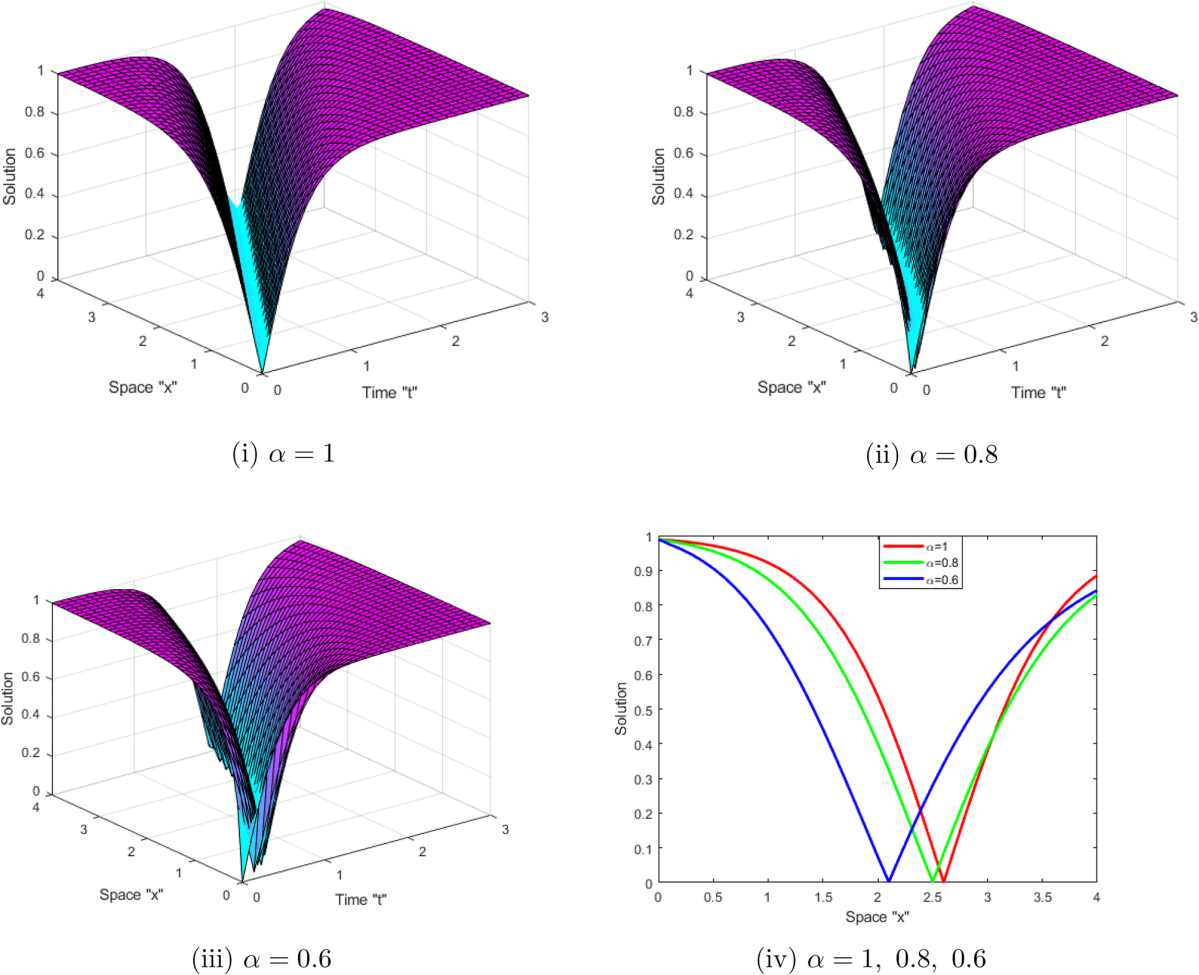
(**i–iii**) 3D-profile of the bright solutions $\left| \mathcal {G}(x,t)\right|$ described in Eq. (15) with $\alpha =1,\ 0.8,\ 0.6$ (**iv**) depict 2D-profile of Eq. (15) with various value of $\alpha$.

Now, we analyze the influence of CD on the obtained solutions of the SFCLLM (1). Suitable values are assigned to the unknown variables to construct a sequence of two- and three-dimensional graphs. Figures 1 and 2 represent the behavior solutions of Eqs. (14) and ( 15), respectively. Figure 1 displays the dark solutions $\left| \mathcal {G}(x,t)\right|$ described in Eq. (14) for $\zeta _{1}=1,\ \zeta _{2}=-2,\ \theta _{1}=\sqrt{2},\ a=b=1,\ \tilde{n} =0.5,\ \rho =0,\ x\in [0,4]$, $t\in [0,3]$ and for $\alpha =1,\ 0.8,\ 0.6$. While, Fig. 2 displays the periodic solutions $\left| \mathcal {G}(x,t)\right|$ described in Eq. (15)for $\zeta _{1}=1,\ \zeta _{2}=-2,\ \theta _{1}=\sqrt{2},\ a=b=1,\ \rho =0,\ x\in [0,4]$, $t\in [0,3]$ and for $$\alpha =1,\ 0.8,\ 0.6.\ $$From these figures, we deduce that when the derivative order $\alpha$ of CD increases, the surface shrinks.

### Impacts of noise

**Figure 3:**
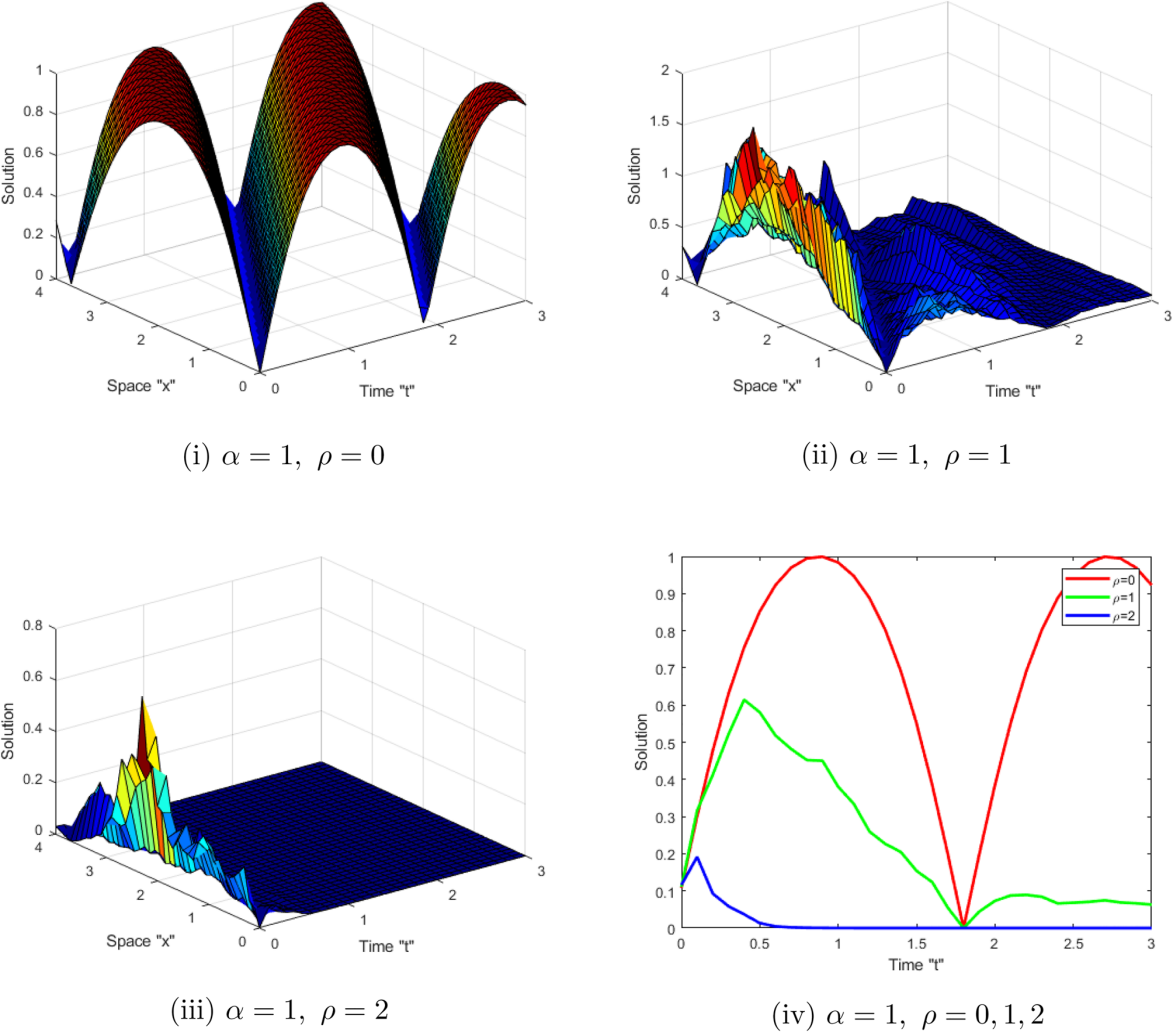
(**i–iii**) 3D-profile of the solution $\left| \mathcal {G}(x,t)\right|$ described in Eq. (14) with $$\alpha =1,\ $$and different $\rho$ (**iv)** depict 2D-profile of Eq. (14).

**Figure 4:**
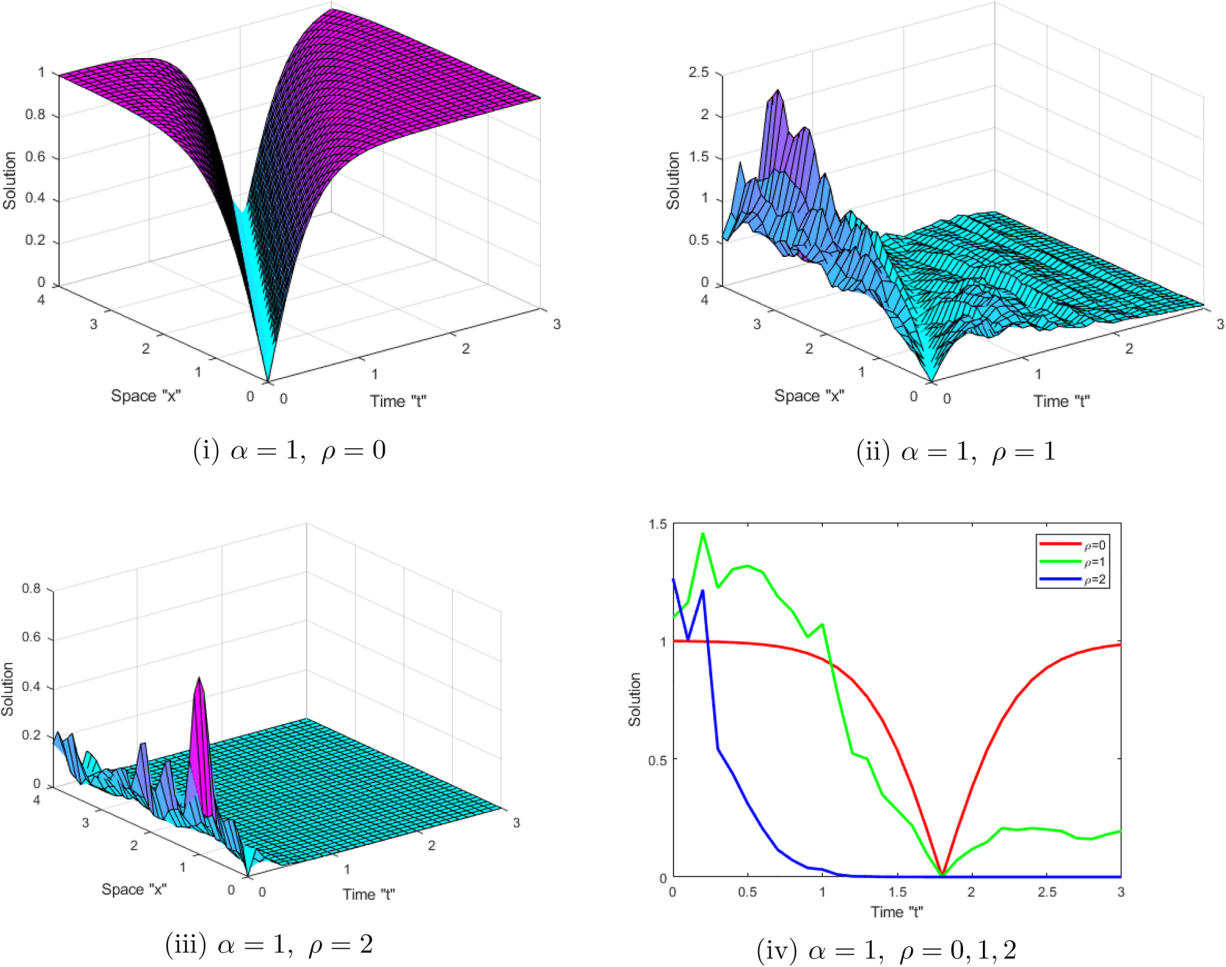
(**i–iii**) 3D-profile of the solution $\left| \mathcal {G}(x,t)\right|$ described in Eq. (15) with $$\alpha =1,\ $$and different $\rho$ (**iv**) depict 2D-profile of Eq. (15).

Now, we study the impact of the time-dependent coefficients on the acquired solutions of the SFCLLM (1). Figure 3 displays the solutions $\left| \mathcal {G}(x,t)\right|$ described in Eq. (14) for $\zeta _{1}=1,\ \zeta _{2}=-2,\ \theta _{1}=\sqrt{2 },\ a=b=1,\ \tilde{n}=0.5,\ \alpha =1,\ x\in [0,4]$, $t\in [0,3]$ and for $\rho =0,\ 1,\ 2$. While, Fig. 4 displays the solutions $\left| \mathcal {G}(x,t)\right|$ described in Eq. (15) for $\zeta _{1}=1,\ \zeta _{2}=-2,\ \theta _{1}=\sqrt{2},\ a=b=1,\ \alpha =1,\ x\in [0,4]$, $t\in [0,3]$ and for $\rho =0,\ 1,\ 2$. From Figs. 3 and 4, we observe that when the noise strength increases, the surface stabilizes around zero.

### Discussion and physical interpretation

This work aimed to get exact solutions of the SFCLLM (1). We used the mapping approach, which yielded a variety of solutions, including periodic solutions, kink solutions, brilliant solutions, dark optical solutions, solitary solutions, and so on. Physically, dark optical soliton denotes waves with lower intensities than the backdrop. Singular solitons are solitary waves with discontinuous derivatives, including compactions with limited (compact) support or peakons with discontinuous first derivatives. These kinds of solitary waves are very important owing to their efficiency and of course flexibility in long distance optical communication. We investigated the influence of conformable derivatives on the obtained solutions and concluded that as the order of fractional derivatives increases, the surface shrinks, as depicted in Figs. 1 and 2. Furthermore, we examined the impact of noise on the solutions and observed that when the noise strength increases, the surface stabilizes around zero as shown in Figs. 3 and 4.

## Conclusions

In this paper, we introduced a large variety of exact solutions to the stochastic fractional Chen Lee Liu model (SFCLLM) (1) forced by multiplicative noise in the Itô sense. By using the mapping approach, rational, elliptic, hyperbolic, and trigonometric stochastic fractional solutions were obtained. These solutions are important for understanding some fundamentally complicated phenomena. The attained solutions are very helpful for applications such as optics, plasma physics and nonlinear quantum mechanics. Finally, we show how the conformable derivative order and the stochastic term affect the exact solution of the SFCLLM (1).

## Data Availability

The datasets used and/or analyzed during the current study are available from the corresponding author upon reasonable request.
